# Social Autonomy and Heteronomy in the Age of ICT: The Digital *Pharmakon* and the (Dis)Empowerment of the General Intellect

**DOI:** 10.1007/s10699-015-9468-1

**Published:** 2015-10-17

**Authors:** Pieter Lemmens

**Affiliations:** Nijmegen, Netherlands

**Keywords:** Digital technologies, Cognitive capitalism, Marxism, Post-autonomism, General intellect, Stiegler

## Abstract

‘The art of living with ICTs (information and communication technologies)’ today not only means finding new ways to cope, interact and create new lifestyles on the basis of the new digital (network) technologies *individually*, as ‘consumer-citizens’. It also means inventing new modes of living, producing and, not in the least place, struggling *collectively*, as workers and producers. As the so-called digital revolution unfolds in the context of a neoliberal cognitive and consumerist capitalism, its ‘innovations’ are predominantly employed to modulate and control both production processes and consumer behavior in view of the overall goal of extracting surplus value. Today, the digital networks overwhelmingly destroy social autonomy, instead engendering increasing social heteronomy and proletarianization. Yet it is these very networks themselves, as technical *pharmaka* in the sense of French ‘technophilosopher’ Bernard Stiegler, that can be employed as no other to struggle against this tendency. This paper briefly explores this possibility by reflecting upon current diagnoses of our ‘technological situation’ by some exemplary post-operaist Marxists from a Stieglerian, pharmacological perspective.

## Introduction

This paper briefly looks at the impact of the new digital network technologies (DNTs) on today’s workers, consumers and citizens ‘living’ with these technologies within the context of today’s cognitive and consumerist capitalism, diagnosing it as predominantly heteronomizing (decreasing the autonomy of subjects) and therefore disempowering, a loud choir of ideological proponents of a bright digital utopia notwithstanding. It also explores, however, how these technologies can nevertheless be recruited to increase social autonomy. More specifically, it explores how these technologies can foster the empowerment of what is called the ‘general intellect’ in the Italian post-Marxist movement of post-autonomism or post-operaism, one of the most vital movements of Marxist thought in recent times. Although this tradition of thinkers, whose ranks include Antonio Negri, Michael Hardt, Paolo Virno, Maurizio Lazzarato and Franco Berardi, is explicitly concerned, among other things, with studying the shifting relationships between technological change on the one side and the class struggle of workers against capital and vice versa on the other, what is generally absent in their analyses is an explicit account of technology and the various ways it mediates, constitutes and conditions social relationships and relations to the self. In short, it lacks an explicit philosophy of technology. In this paper, therefore, I will use Bernard Stiegler’s pharmacological conception of technology and see how it can be used to elaborate and enrich the analyses of today’s general intellect by the operaist-autonomist tradition, which always revolve around the issue of recomposition (an autonomist term referring to the unification, politicization and becoming-conscious of itself—i.e., *für sich* and not just *an sich*—of the labor force) or the empowerment of the general intellect as a process of collective political subjectivation (the process of becoming a political subject) of workers/citizens. Given the exploratory nature of this paper, I will only provide some suggestions in that direction.

## Conceptual Preliminaries: Cognitive Capitalism and the General Intellect

Cognitive capitalism, a term coined by the French economist Yann Moulier-Boutang, refers to the current mode of capitalism in which not labor power but cognitive and affective capacities are the crucial determinants of productivity and the main sources of surplus value. It is a capitalism that thrives on the exploitation of cognition, attention, affection and what Moulier-Boutang calls attention-power and invention-power (Moulier-Boutang 2011, 32). It involves the capturing of monetary gains from knowledge production and innovation by a ‘collective intelligence’ or a ‘general intellect’ described by Lazzarato in terms of ‘cooperation between brains’ (Lazzarato 2002). Using the metaphor of bees, Moulier-Boutang writes that the wealth produced under cognitive capitalism derives from the constant ‘pollination’ of society by the multitudes on the wings of the digital networks (Moulier-Boutang 2011, 108). Cognitive capitalism submits cognition to the economic imperatives of profit maximization and competition, and replaces industrial capitalism, which was mainly based on the exploitation of labor power as muscular capacity. In the context of cognitive capitalism, and as a result of the deployment of DNTs, the human cognitive apparatus is increasingly incorporated into the digital cognosphere.

Another central concept within post-autonomist discourse, derived from the famous ‘Fragment on machines’ in Karl Marx’ *Grundrisse* and reinterpreted in the context of today’s cognitive capitalism, is that of the general intellect. Marx uses this notion of the general intellect in the *Grundrisse* only once, to designate the objectified labor incorporated in machines or as the power of knowledge and science, incarnated in machines as dead labor or as fixed capital subsuming and controlling variable capital or living labor (i.e. workers). For Marx, the notion of general intellect refers to the system of machinery as the scientifically objectified power of labor that is transformed *as such* into the power of capital. In the machinery of industrial capitalism, the laborers’ knowledge appears as something alien and external to him and living labor appears as subsumed under self-activating objectified labor. As Marx writes: ‘It belongs to the concept of capital that the increased productive force of labor is posited rather as the increase of a force outside itself, and as labor’s own debilitation’ (Marx 1993, 702).

In post-autonomism, however, the concept of the general intellect is understood quite differently, not as external machinery opposing living labor as fixed capital but as fixed capital *inside* living labor, i.e., as an attribute of living labor. In cognitive capitalism, the general intellect comes to refer to the information, the communication, the languages, codes, skills and competences of the immaterial laborers. As Antonio Negri emphasizes, cognitive capitalism’s labor force carries its means of production once again inside itself, in its brains (Negri 2008b, 66). It is fixed capital residing in the nervous systems of living labor and is referred to in the HR departments of companies and public institutions as ‘human capital’. According to Negri and many other post-autonomists, this general intellect as the fixed capital residing in human brains grants labor a relative yet increasing independence with respect to capital and allows it a growing margin of autonomy (ibid.). It would also represent a break with the process of proletarianization, since it involves a certain reappropriation of the means of production by labor, thereby putting an end to the dialectics of the instrument of labor first theorized by Hegel (Negri 2008a, 65). As will be shown, however, this assertion is highly doubtful.

## Digital Network Technologies in Post-Autonomist Thought

Post-autonomists generally applaud the digital revolution and the chances it provides for the struggle of labor against capitalist domination. Moulier-Boutang, and in particular Hardt and Negri in their *Empire* trilogy, affirm that DNTs are ultimately favorable to the expansion of the autonomy of the multitude vis à vis capital, although they enable new and highly effective forms of labor control as well (Moulier-Boutang, Ch. 4, Hardt and Negri 2000, 294–7). Both labor and capital have to operate under the network condition today, the digital networks providing the matrix for both the new figure of sovereignty known as empire and of immaterial production by the multitude, the living prey of *and* alternative to empire. For Hardt and Negri, given its globally networked condition, the multitude as the assemblage of singularities forming the new proletariat is principally capable of self-rule and autonomy, since it is able to produce its own means of production (that is to say: cooperation and communication as the substance of the common) all by itself, independent of capital (Hardt and Negri 2009, 140f). In fact, they suggest that the immaterial production and decision-making via DNTs by the multitude provides, by itself, the model for a genuine multitudinous and absolute democracy (Hardt and Negri 2004, 339). So, for Hardt and Negri, DNTs are ultimately empowering and autonomizing, providing, in principle, the socio-technological condition for a global radical democracy as the self-rule and self-management of the multitude.

In stark contrast to Hardt and Negri, Franco Berardi fails to perceive the emancipatory potential of DNTs. His term for the contemporary form of capitalism is semiocapitalism: a capitalism thriving on the exploitation of linguistic, symbolic and cognitive, that is to say sign-producing or semiotic labor (Berardi 2009a, 21). Berardi points to the fact that cognitive or immaterial labor, far from gaining more and more autonomy with respect to capital, is becoming increasingly captured and controlled *inside* the digital networks, through the generalized implementation of what he calls technical and financial *automatisms* operating exclusively according to the logic of competition and surplus extraction and engendering more and more psychological and social automatisms (ibid., 88; Berardi 2009b, 7).

What he sees emerging in the context of cognitive capitalism is an increasing integration of the human mind into the digital circuits of capital, the organic nervous systems of the cognitariat more and more incorporated into and submitted to the operational logics and rhythms of the digital nervous system, shaping a genuine ‘assembly line of net-production’ (Berardi 2011, 55). The cognitariat’s consciousnesses are exposed to an ever more expanding and accelerating cogno- and infosphere, a situation that leads to a growing discrepancy between cyber*space*, the ever growing and virtually infinite mass of semiotic and informational commodities circulating on the net, and cyber*time*, the finite mental time necessary for processing and elaborating this information (Berardi 2009b, 40–1). This discrepancy induces all kinds of psychopathologies—like depression, anxiety, panic and attention disorders of all kinds—and leads to the exhaustion and ultimately destruction of psychic and mental energies (Berardi 2009a, 158). It erodes subjects’ sensibility and affectivity due to the constant pressure to adapt one’s psychic apparatus to the codes and rhythms of the network.

Moreover, the constant mobilization of attention as well as the fragmentation and fractalization of workers’ time prevents the formation of collectivity and solidarity as a necessary basis for effective resistance and action against the domination of capital. It is due to these paralyzing and sterilizing labor conditions, Berardi claims, that the political recomposition of the general intellect, and the possibilities for political subjectivation and the creation of solidarity, is systematically frustrated (Berardi 2011, 14). The necessity of always ‘being connected’ for participating in cognitive production supposes the adjustment of one’s psychic apparatus to the codes, the logic, the speed and the rhythm of the infosphere and engenders the loss of reflexivity and the erosion of sensibility and receptiveness.

A central theme in the admittedly quite gloomy analyses of Berardi is that of the exhaustion of libidinal energies as a defining dimension of contemporary cognitive labor, a theme that also runs through the work of Stiegler, as we will see. The minds of the cognitarians, glued to demanding screens from the morning until the evening, are more and more disconnected from their bodies as well as from the social body. Berardi states that it cannot be denied that the intellectual capacity of today’s general intellect is potentially boundless—and here he is largely in agreement with Hardt and Negri and Moulier-Boutang—but that it currently lacks any consciousness of itself (ibid., 163). The creation of such a self-consciousness is precisely the emancipatory political task of the future.

Berardi’s work is important in that it shows that transformations in the techno-cognitive environment change the conditions for political subjectivation and redefine the possibilities as well as the constraints for collective struggle. However, he only seems to perceive the negative side of current transformations. For him, the big political question is how to create a self-consciousness out of the general intellect so as to gain a common ground of understanding for collective action that can be effective and truly autonomizing. His suggestion is that the kind of politics necessary for such a task of commonization and autonomization should have the character of a *therapy* and not of political resistance and action in the traditional, say Leninist sense of the term, which would only deepen our exhaustion and depression, as he argues (ibid., 37–8). An effective and vitalizing future emancipatory politics should concern itself instead with re-directing the social investments of desire, from the pathogenic investment in competition, accumulation and work to an investment in community, friendship and love. At some point he even proposes the embracement of a ‘radical passivity’ as the only way to counter the neoliberal ethos of relentless competition and productivity (ibid., 138).

Thus, for Berardi, desire and affect are crucial. Important to note here is that Berardi conceives of desire not anymore (as Marcuse, Foucault and also Deleuze and Guattari were inclined to do in their days) as a positive force that is inherently emancipatory but, inspired by Jean Baudrillard, as a field upon which conflicting forces intersect, principally mediated by technology (Berardi 2009a, 154). The problem with Berardi, however, is that no effort is made of developing a strategy for confronting and dealing with the diagnosed situation in terms of that situation itself, i.e., on the terrain of DNTs, the inevitable condition for political engagement in our time. Berardi keeps silent when it comes to the question of how to transform the situation from within, so to speak. He does not seem to perceive any chance of changing the currently hegemonic disempowering efficacy of the DNTs for the better, or at least he does not make any effort in theorizing such a possibility. This might be explained from the fact that he lacks a theory of human-technology interaction and of technological change. Neither does he possess any conception of a technopolitics. For this, we have to turn our attention to the work of Stiegler.

## Bernard Stiegler: Pharmacology of the Digital

For Stiegler, the DNTs represent nothing less than an epochal technological transmutation, that is to say a mnemotechnological (concerning the technologies of memorization and information and communication more generally) transmutation no less decisive and disruptive than the invention of writing at the dawn of Western civilization and printing at the dawn of modernity. It represents a new, that is to say third phase in what the French linguist Sylvain Auroux calls grammatization, as the process of formalization and externalization of human language, the two earlier phases being that of alphabetization and writing (Stiegler 2014a, 54). As such, DNTs usher in a wholly new *episteme* in the sense of Michel Foucault and allow for totally new epistemologies in the sense of Gaston Bachelard (Stiegler 2014b, 148). They will totally reconfigure the human mindset, like writing did in ancient Greece at the time of Plato, as classicists like Ong, Goody and Havelock have shown (Ong 2012; Goody 1977; Havelock 1986), or like printing did from the early fifteenth century onwards, as Elisabeth Eisenstein has pointed out (Eisenstein 2012). They are also on the way of reconfiguring our neuronal structures, changing our brain structure, as neurologist and psycholinguist Maryanne Wolf has argued, from ‘reading brains’ to ‘digital brains’, just like writing turned the ‘oral brains’ of our pre-literate ancestors into ‘reading brains’ (Wolf 2010). More directly, they also furnish a new ‘political organology’ (see below) that has yet to be ‘appropriated’ politically.

Stiegler has developed an *organological* theory of human, technology and society interaction, which I will sketch very briefly here. This *general* organology conceives of human evolution and history as technogenic processes involving three organ systems: (1) psychosomatic organs, (2) social organizations and (3) technical organs (Stiegler 2013a, b, c, 419–20). Changes in the technical organs always induce de-functionalizations and subsequent re-functionalizations in the psychosomatic and social organs, which—in the course of evolution and later during history—are involved in a constant process of adoption of (or adaptation to) new technologies. The relations between these three organ systems are of a transductive nature, meaning that they only take shape within and from out of their relation to each other.

What is most important in the current context is that the relations between these three organ systems constitute circuits of desire, of libidinal energy, given that the technical organs and the social organizations give shape to the drives residing in the psychosomatic organs (Stiegler 2013b, 320). Now, the technical organs, Stiegler argues, can *both* intensify the binding of drives into libidinal energy, supporting processes of sublimation and psychic elevation, *and* cause their decomposition, which leads to desublimation and psychic regression. As such, every organology constitutes a libidinal economy. A *political* organology would consider the conditioning effects of the technical organs, or in other words the technical milieu, on the formation of political affects, most importantly on what Aristotle in his political writings called *philia*, or collective desire, as the conditio sine qua non of all political life.

Now, for Stiegler, the fact that technical organs, i.e., technologies of all sorts but most explicitly so-called mnemo- or psychotechnologies, can both positively and negatively condition libidinal economies, is related to the fact that they are what he calls *pharmaka*, a Greek word that means both poison and medicine. As compensations for the ‘original lack’ [*défaut originaire*] of intrinsic attributes characteristic of human beings, technical *pharmaka* can both aid and impede, both support and undermine aspects of those beings. They are both toxifying and curative. That is to say: given the fact that they are constitutive of the human being’s thrown and projective being-in-the-world, they have an autonomizing as well as a heteronomizing potential (Stiegler 2013a, 421–2). This pharmacological conception of technologies has affinities with Andrew Feenberg’s idea of technical ambiguity (Feenberg 2002, 53) but is more firmly rooted in an ‘onto-anthropological’ understanding of what the human life form as a technical life form *is*.

## Technopolitics as Pharmako-Therapy

As instruments for political subjectivation, technical *pharmaka* like DNTs can both support politicization and depoliticization, both emancipation and docilization. They can both elevate and ‘dumb down’ collective intelligence by supporting either short circuits (‘egoistic’ circuits of drive) or long circuits of individuation (producing desire and sociality). What DNTs ‘are’—and here Stiegler is in agreement with Feenberg—depends to a large extent on how they are adopted by individuals and collectives. It may be argued that Berardi’s diagnosis exclusively emphasizes the proliferation of short circuits that exhaust libidinal energy and induce drive-based behavior (i.e., capturing libidinal energy into short loops of immediate gratification that tendentially induce frustration in subjects), thereby acknowledging only the toxic tendency and disregarding or at least neglecting any *thought* of a therapy—other than that of creating zones of human resistance outside of the ‘system’—that would precisely be based on an emancipatory and autonomizing adoption or appropriation of DNTs that would counter its toxic, heteronomizing tendencies.

Stiegler stresses that autonomy exists only in an intimate relation with technical heteronomy, on the condition, that is, that it is adopted intelligently, reflexively and with care, which assumes a practice, that is to say a *therapy*, a *socio*therapy, or in short: a politics (Stiegler 2013c, 41, 45). DNTs, as Stiegler argues again and again in all of his writings, can truly function as a technical milieu creating long circuits and supporting a new social autonomy. This presupposes, however, a collective appropriation of that milieu, so that it becomes an associated milieu instead of a dissociated milieu, the latter being the dominant situation today. In fact, DNTs are ideally suited for this, certainly so if compared to the older analog information and communication technologies like radio and television. To give some idea of that: DNT’s are characterized by multilinearity, which allows for demassification and desynchronized access (think of podcasts for instance), by annotability, which allows for tagging and the bottom-up creation of metadata, by hypermediality, which means that they can handle all levels of grammatization from texts to genomic sequences, and most importantly by bidirectionality, which allows receivers to be senders and vice versa (Stiegler 2006, 219).

Stiegler’s assessment of the current impact of DNTs on the general intellect, however, is closer to the view of Berardi than to those of Hardt and Negri, for instance. He is much less pessimistic however. In fact, the DNTs are for Stiegler nothing less than the way out of the state of deep intoxication that our current mnemotechnical milieu suffers from as a result of its annexation by consumerist and cultural capitalism. It sometimes even looks as if DNTs represent, for him, a kind of Heideggerian saving power, if only we were able to succeed in carrying through what he refers to sometimes as a ‘pharmacological turn’ of this *pharmakon*.

Concurring with the analysis of Berardi and fundamentally disagreeing with the views of Hardt and Negri, however, Stiegler contends that instead of tendentially autonomizing and empowering the multitude because of the increasing independence of the general intellect from capitalist control, in reality the general intellect appears to be the object of an intense process of *proletarianization* today that seems to disempower it and rob it of its autonomy, producing a regression of the collective intelligence of the general intellect that he refers to as a state of ‘systemic stupidity’ (Stiegler 2010, 47).

Instead of fixed capital migrating to living labor (the immaterial means of production increasingly residing in the brains of cognitive laborers), thereby granting it more autonomy as Hardt and Negri claim, Stiegler states that through generalized automation as exteriorization and short-circuiting of psychic and cognitive functions in the DNT, we cannot but admit that the multitude’s collective intelligence is becoming increasingly proletarianized (ibid., 45). Instead of a growing commonization and autonomization of the multitude, what seems to be the rule nowadays is cognitive proletarianization and psychic heteronomization of the singularities that make up the multitude. So-called cognitive capitalism in fact destroys cognition, forcing workers to adapt their cognitive apparatus and capacities to a digital technical milieu that ruthlessly imposes the imperatives of profit and competition. It is true that DNTs enable the ‘cooperation between brains’ (Lazzarato) but so far they have predominantly served capital in controlling this cooperation and capturing its fruits (ibid., 47).

However, this is a pharmacological situation and as such it is anything but hopeless. While still functioning largely as technologies of capture and control (as analyzed by Berardi), at the same time the DNTs are gradually beginning to be transformed into the conditions for the emergence of a wholly new associated milieu that can function as the support of a therapeutic countermovement able to fight the proletarianizing tendency. Phenomena like Free Software, peer-to-peer, Wikipedia, Wikileaks, Anonymous, and many other commons-based collaborative projects can be understood as being the first movements in that direction, movements that are characterized by Stiegler in terms of deproletarianization. The hackers and their anti-protestant—although not necessarily anti-capitalist—ethics of voluntary cooperation, sharing, passion and care with respect to digital work (as expounded by Pekka Himanen in his well-known bestseller *The Hacker Ethic*) are the exemplary heroes of this countermovement (Himanen 2001).

## The Need for a Class-Based Pharmacology of the Digital

What is lacking in Stiegler, however, is a class-based analysis of this pharmacological situation in terms of an account of the possibilities for recomposition of the general intellect. Such an account should be developed, I claim, because it seems unrealistic if not totally illusory to put too much trust, as Stiegler does in his writings, in the initiative of governments or representatives of today’s public institutions, to promote and support the emergence of alternative modes of production and collaboration beyond the consumerist model, that can break with the current hegemony (Stiegler 2006). In an all-out class war of capital and the state against the laboring multitudes, which is currently waged with an unprecedented brutality and cynicism, calling for a public power to positively intervene in the situation and prescribing a sociotherapy in terms of a positive pharmacology seems more than a bit naive. Such a therapeutic project should and can only emerge from ‘below’, i.e., from the multitude itself, or more realistically from its dissident and least proletarianized factions.

Unfortunately, there is no space here to embark on a compositionalist pharmacological analysis of today’s DNTs. For that reason I will necessarily have to limit myself here to proposing a few questions that could guide such an analysis. It is clear that DNTs by themselves, left within the confines of the market economy, will never bring about true social autonomy, but only enforce the toxic, desublimatory, heteronomizing tendency that Berardi and also Stiegler rightly diagnose. What is needed is a political project of adoption in terms of a therapy and this ‘therapy’ must intervene at a systemic level and aim for a new organization of society, no less! A society that would be truly *intelligent* and *autonomous* in the sense that it, as the late André Gorz put it nicely in one of his books, paraphrasing a famous dictum by Karl Marx, should create the conditions ‘in which the full development of each person’s ability is everyone’s aim’ (Gorz 2010, 109) and in which production would serve human development instead of human development serving the production of surplus value (ibid., 113).

In light of the critical diagnoses offered by Berardi and Stiegler, the following questions could lead a pharmacological analysis of class composition in the current situation. First of all: how can workers and ‘deconsumerizing’ citizens employ the digital *pharmakon* *itself* against the hegemonic tendencies it now supports? How can DNTs be appropriated or maybe newly designed so that they can be used to struggle against the constant acceleration of online life—and as a consequence also of offline life—that they now everywhere support? How can they be repossessed for the purpose of ‘recapacitation’ (Amartya Sen) of the cognitariat as the subject of the general intellect? How can the digital *pharmaka* be changed so as to slow down life and shape the conditions and the time for reflection, critique and a more free and autonomous disposition of our individual and collective time? How can the digital *pharmaka* be changed so as to foster commonality and solidarity instead of atomizing and individualizing us (think also of social networks like Facebook and Myspace here)? How can we change the underlying algorithms—the technical codes so to speak (not necessarily but possibly in the sense of Feenberg)—that constitute the technofinancial automatisms servant to capital that Berardi writes about? How can the digital *pharmaka* be appropriated or redesigned so as to become the supportive technologies of what Stiegler has called an ‘otium of the people’, i.e., as technologies of the self and others, of care of the self and care of the other in the sense of Michel Foucault, instead of promoting competition and increasing automation, heteronomization and carelessness? How can these *pharmaka* be creatively re-appropriated to struggle against the disaffection, disaffectation and libidinal exhaustion and how can they become the instruments of new modes of critical and ‘deep’ attention? How can they become inducive to/engaged in processes of commonization and the formation of ‘collective desire for the collective’, instead of increasing individualization? How can they be remade to reconnect subjectivities with the social fabric they still remain part of yet hardly experience anymore?

All these questions suppose that we should not only learn the ‘art of living with ICTs (information and communication technologies)’, but that we should also learn to fight, produce, commonize and *live in common*, i.e., collectively manage our collective existence, with ICTs. ‘The art of living with ICTs’ should in any case not be understood in an adaptationist sense, as the necessity of having to cope with something inevitable, of having to get along with it, of having to adapt ourselves to it. Instead we should try to adapt those technologies to what *we*, as socio-technical creatures, collectively decide to be a life *worth* living—i.e., in the sense of *adopting* and creatively appropriating them as weapons (in the sense of Gilles Deleuze) for (re-)conquering our collective autonomy in the face of an increasingly totalitarian capitalism bent on controlling subjects more and more through what Lazzarato has most recently described as ‘machinic enslavement’ (Lazzarato 2014, 23–38). That is the most urgent question, I would like to suggest, with respect to the question of the ‘art of living with ICTs’.
